# Supplementary material for: Analysis of PPARγ Signaling Activity in Psoriasis
Source: Int J Mol Sci. 2021 Aug 10;22(16):8603. doi: 10.3390/ijms22168603 (PMC8395241; doi:10.3390/ijms22168603)
Supplement: Supplementary file 1 [file ijms-22-08603-s001.zip › Supplemental materials_Analysis of PPARg signaling activity in psoriasis/Pathway models/Models images and html files/Differentiation of psoriatic T cells/6379.html]

IL13


# Protein IL13

|  |  |
| --- | --- |
| URN | urn:agi-llid:3596 |
| Total Entities | 0 |
| Connectivity | 5590 |
| Name | IL13 |
| Description | interleukin 13 |
| Notes | This gene encodes an immunoregulatory cytokine produced primarily by activated Th2 cells. This cytokine is involved in several stages of B-cell maturation and differentiation. It up-regulates CD23 and MHC class II expression, and promotes IgE isotype switching of B cells. This cytokine down-regulates macrophage activity, thereby inhibits the production of pro-inflammatory cytokines and chemokines. This cytokine is found to be critical to the pathogenesis of allergen-induced asthma but operates through mechanisms independent of IgE and eosinophils. This gene, IL3, IL5, IL4, and CSF2 form a cytokine gene cluster on chromosome 5q, with this gene particularly close to IL4. [provided by RefSeq, Jul 2008] |
| Primary Cell Localization | Extracellular |
| Class | Ligand |

---

|  |  |
| --- | --- |
| Pathway | Immunoglobulin Class-Switch Recombination Activation |
|  | Immunoglobulin Genes Transcriptional Activation |
|  | T-Cell Maturation (Hypothesis) |
|  | Th2-Cell Differentiation |
|  | Lipoxins and Resolvins in Inflammation Resolution |
|  | Basophil Activation |
|  | Eosinophil Survival by Cytokine Signaling |
|  | Eosinophil Activation |
|  | Mast-Cell Activation without Degranulation |
|  | Mast-Cell Activation without Degranulation through IL33/IL1RL1 Signaling |
|  | Mast-Cells De Novo Synthesized Mediators via IgE Independent Signaling |
|  | anti-Inflammatory Function of Macrophage M2 Lineage |
|  | Proteins Involved in Atherosclerosis |
|  | Proteins Involved in Myocarditis |
|  | Mast-Cells Activation in Atopic Dermatitis |
|  | Genes with Mutations Associated with Atopic Dermatitis |
|  | Acute Phase in Atopic Dermatitis |
|  | Proteins Involved in Atopic Dermatitis |
|  | Atopic Dermatitis |
|  | Genes with Mutations Associated with Psoriasis |
|  | T-Cells Differentiation Block in Psoriasis |
|  | Proteins with Altered Expression in Psoriasis |
|  | Th2-Cell Function in Systemic Lupus Erythematosus |
|  | Proteins Involved in Diabetes Mellitus Type 1 |
|  | B-Cell Activation in Crohn's Disease |
|  | Proteins Involved in Helicobacter Infections |
|  | Leukocyte Migration toward the Endothelial Cell in Ulcerative Colitis |
|  | Proteins Involved in Ulcerative Colitis |
|  | Th2-Cell Function in Ulcerative Colitis |
|  | T-Cell Cytotoxic Response in Ulcerative Colitis |
|  | Diffuse Large-B-Cell Lymphoma |
|  | Primary Mediastinal B-cell Lymphoma (PMBL) Subtype |
|  | Proteins Involved in Hodgkin Lymphoma |
|  | Hodgkin Lymphoma |
|  | Hodgkin and Reed-Sternberg Cells Reprogramming |
|  | Proteins Involved in Pancreatic Cancer |
|  | ADAM33 Role in Asthma |
|  | Eosinophil Survival in Asthma |
|  | IL33 Signaling Related Eosinophilia in Asthma |
|  | TSLP Signaling in Bronchial Epithelial Cell in Asthma |
|  | Periostin (POSTN) Production by Airway Epithelium in Asthma |
|  | IgE Induces Airway Smooth Muscle Cell Proliferation |
|  | Airway Smooth Muscle Cell Contraction |
|  | Goblet-Cell Exocytosis in Asthma |
|  | Goblet-Cell Related Mucus Secretion in Asthma |
|  | Club-Cell Role in Asthma |
|  | Basophil Activation in Asthma |
|  | B-Cell Mediated IgE Production in Asthma |
|  | Th2-Cell Response in Asthma |
|  | Mucin Production in Goblet Airway Epithelial Cells |
|  | Th17-Cell and Th1 Immune Responsein Psoriatic Arthritis |
|  | Keratinocyte Activation in Psoriatic Arthritis |
|  | Proteins Involved in Psoriatic Arthritis |
|  | B-Cells Function in Systemic Scleroderma |
|  | Th2-Cells Function in Systemic Scleroderma |
|  | Genes with Mutations Associated with Systemic Scleroderma |
|  | Proteins Involved in Systemic Scleroderma |
|  | Proteins with Altered Expression in Systemic Scleroderma |
|  | Proteins Involved in HIV Type 1 Infection |
|  | CD4+ T-Cell Function Decline in HIV |
|  | Genes with Mutations Associated with Psoriatic Arthritis |
|  | Proteins Involved in Glioblastoma |
|  | Proteins Involved in Glioma |
|  | Proteins Involved in Endometriosis |
|  | Genes with Mutations Associated with Asthma |
|  | Proteins with Altered Expression in Asthma |
|  | Proteins Involved in Chronic Obstructive Pulmonary Disease |
|  | Proteins Involved in Pulmonary Emphysema |
|  | Mucin Hyperproduction in Goblet and Mucous Cells |
|  | Proteins Involved in Chronic Bronchitis |
|  | Proteins with Altered Expression in Cystic Fibrosis |
|  | Proteins Involved in Polycystic Kidney Disease |
|  | Epinephrine/Gs Expression Targets |
|  | Epinephrine/Gq Expression Targets |
|  | IL13/STAT6/ZEB1 Signalling Promotes Cancer Metastatic Progression |
|  | Tumor Infiltrating Macrophages in Cancer Progression and Immune Escape |
|  | Myeloid Derived Suppressor Cells in Cancer Immune Escape |
|  | Proteins with Altered Expression in Cancer Immune Escape |
|  | Insulin -> STAT Expression Targets |
|  | Insulin -> CEBPA/CTNNB/FOXA/FOXO Expression Targets |
|  | Insulin -> MEF/MYOD Expression Targets |
|  | Insulin -> ELK/SRF/HIF1A/MYC/SREBF Expression Targets |
|  | IL13R -> STAT6 Signaling |
|  | IL13R -> STAT Signaling |
|  | IL13 Expression Targets |
|  | IL3 Expression Targets |
|  | IL2 Expression Targets |
|  | LPA Expression Targets |
|  | F2R/F2RL1 -> Expression Targets in Lymphoid System and Blood |
|  | TNF Receptors -> Expression Targets in Lymphoid System and Blood |
|  | Elevated Receptors -> Expression Targets in Thymus |
|  | Adenosine Expression Targets |
|  | KITLG -> STAT Expression Targets |
|  | KITLG -> AP-1/CREB/CREBBP/MYC Expression Targets |
|  | TCR -> NFAT Expression Targets |
|  | TCR -> AP-1 Expression Targets |
|  | TLR4 -> AP-1 Expression Targets |
|  | IL1B -> PGE2 Expression Targets |
|  | IL1B Expression Targets |
|  | PPAR Psoriasis |
|  | Model of PPARG signaling in psoriasis |
|  | Overview. Diseases of the respiratory (chapter 9) |
|  | 2\_Acute phase initiation in atopic dermatitis |
|  | 1\_Differentiation of psoriatic T cells |
|  | 1\_Th2 cell response in asthma |
|  | 3\_2\_Regulation of airway surface liquid (ASL) thickness |
|  | 3\_1\_Mucus accumulation in asthma |
|  | 2\_2\_Eosinophilia and neutrophilia in asthma: eosinophil apoptosis |
|  | 4\_2\_Airway smooth muscle cells proliferation and airway tissue remodeling |
|  | 4\_Mucin hyperproduction in goblet and mucous cells in COPD |
|  | 3\_Leukocyte migration toward the endothelial cells in the intestine microvasculature |
|  | 2\_Intestine epithelial cells drive immunological responses in ulcerative colitis |
|  | 4\_1\_Polymorphisms associated with inflammatory bowel diseases |
|  | drug-target moltransport |
|  | drug-target molsynthesis |
|  | Differentiation of psoriatic T cells |

---

|  |  |
| --- | --- |
| Group | Genes with Mutations Associated with Atopic Dermatitis |
|  | Genes with Mutations Associated with Psoriasis |
|  | Genes with Mutations Associated with Graves Disease |
|  | Genes with Mutations Associated with Asthma |
|  | Genes with Mutations in GWA Studies of Asthma |
|  | Genes with Mutations Associated with Psoriatic Arthritis |
|  | Genes with Mutations Associated with Systemic Scleroderma |

---

|  |  |
| --- | --- |
| Source | curated |
|  | GO component |

---

|  |  |
| --- | --- |
| MedScan ID | 3596 |

---

|  |  |
| --- | --- |
| LocusLink ID | 3596 |
|  | 16163 |
|  | 116553 |
|  | 281247 |

---

|  |  |
| --- | --- |
| Alias | interleukin IL-13 cytotoxin |
|  | T-cell activation protein P600 |
|  | interleukin 13 |
|  | IL13\_HUMAN |
|  | interleukin IL13 |
|  | NC30 |
|  | IL-13 cytotoxin |
|  | RP23-239O19.1 |
|  | interleukin-13 receptor ligand |
|  | IL13 |
|  | IL XIIIs |
|  | OTTMUSP00000005816 |
|  | interleukin IL13\_HUMAN |
|  | MGC116789 |
|  | MGC116786 |
|  | IL XIII |
|  | interleukin IL XIII |
|  | interleukin IL XIIIs |
|  | MGC116788 |
|  | LOC96500 |
|  | P600 |
|  | IL-13 |
|  | interleukin-13 |
|  | ALRH |
|  | BHR1 |
|  | allergic rhinitis |
|  | Bronchial hyperresponsiveness-1 (bronchial asthma) |

---

|  |  |
| --- | --- |
| GO ID | 0005125 |
|  | 0005144 |
|  | 0071260 |
|  | 0019221 |
|  | 0006955 |
|  | 0006954 |
|  | 0001774 |
|  | 0033861 |
|  | 1903660 |
|  | 2000352 |
|  | 1901247 |
|  | 1901215 |
|  | 0071635 |
|  | 0030890 |
|  | 0120162 |
|  | 0032723 |
|  | 0010628 |
|  | 0002639 |
|  | 1901251 |
|  | 0043032 |
|  | 0043306 |
|  | 2000231 |
|  | 0050714 |
|  | 0051281 |
|  | 0048661 |
|  | 0042531 |
|  | 0010155 |
|  | 0045471 |
|  | 0032496 |
|  | 0035094 |
|  | 0005737 |
|  | 0009897 |
|  | 0005576 |
|  | 0005615 |
|  | 0005126 |
|  | 0071345 |
|  | 0043270 |
|  | 0010469 |
|  | 0009612 |
|  | 0005623 |
|  | 0007267 |
|  | 0006928 |
|  | 0042526 |
|  | 0007165 |
|  | 0008284 |
|  | 0005625 |
|  | 0005515 |
|  | 0004871 |
|  | 0008283 |
|  | 0008009 |
|  | 0019735 |

---

|  |  |
| --- | --- |
| KEGG ID | hsa:3596 |
|  | mmu:16163 |
|  | rno:116553 |

---

|  |  |
| --- | --- |
| MedLine Reference | 10331006 |
|  | 12162438 |
|  | 12919086 |
|  | 15042582 |
|  | 15961726 |
|  | 16522370 |
|  | 7916615 |
|  | 10079098 |
|  | 10330435 |
|  | 10358179 |
|  | 10491413 |
|  | 10508526 |
|  | 10655497 |
|  | 10679097 |
|  | 10704283 |
|  | 10920230 |
|  | 10940898 |
|  | 10943842 |
|  | 10992460 |
|  | 10995789 |
|  | 11032397 |
|  | 11070175 |
|  | 11120785 |
|  | 11323701 |
|  | 11331286 |
|  | 11441116 |
|  | 11441125 |
|  | 11466392 |
|  | 11481612 |
|  | 11591797 |
|  | 11694459 |
|  | 11714822 |
|  | 11714828 |
|  | 11737071 |
|  | 11745342 |
|  | 11830478 |
|  | 11831873 |
|  | 11884467 |
|  | 11893731 |
|  | 12045241 |
|  | 12091879 |
|  | 12107190 |
|  | 12150887 |
|  | 12355446 |
|  | 12356575 |
|  | 12370375 |
|  | 12397019 |
|  | 12438750 |
|  | 12444158 |
|  | 12466851 |
|  | 12525563 |
|  | 12540780 |
|  | 12568496 |
|  | 12574343 |
|  | 12594274 |
|  | 12642602 |
|  | 12648490 |
|  | 12669034 |
|  | 12707030 |
|  | 12842850 |
|  | 12847266 |
|  | 12874364 |
|  | 12897202 |
|  | 12941477 |
|  | 14568929 |
|  | 14707083 |
|  | 14734772 |
|  | 14764728 |
|  | 14970180 |
|  | 15034018 |
|  | 15087305 |
|  | 15128831 |
|  | 15192232 |
|  | 15229217 |
|  | 15240714 |
|  | 15240716 |
|  | 15272080 |
|  | 15356151 |
|  | 15378055 |
|  | 15470039 |
|  | 15494506 |
|  | 15507491 |
|  | 15563687 |
|  | 15564479 |
|  | 15618138 |
|  | 15634931 |
|  | 15647285 |
|  | 15741272 |
|  | 15814686 |
|  | 15816016 |
|  | 15944273 |
|  | 15944318 |
|  | 16000330 |
|  | 16034131 |
|  | 16039582 |
|  | 16081840 |
|  | 16116211 |
|  | 16239566 |
|  | 16251181 |
|  | 16317093 |
|  | 16327802 |
|  | 16339553 |
|  | 16339565 |
|  | 16439363 |
|  | 16478801 |
|  | 16493031 |
|  | 16517724 |
|  | 16549365 |
|  | 16552085 |
|  | 16568498 |
|  | 16585593 |
|  | 16598817 |
|  | 16702603 |
|  | 16816360 |
|  | 16893457 |
|  | 16988272 |
|  | 17035062 |
|  | 17088137 |
|  | 17148674 |
|  | 2521353 |
|  | 7903102 |
|  | 7907877 |
|  | 8096327 |
|  | 8099936 |
|  | 8605347 |
|  | 8871614 |
|  | 9162077 |
|  | 9177770 |
|  | 9512421 |
|  | 9531306 |
|  | 9558109 |
|  | 9619371 |
|  | 9635196 |
|  | 9768762 |
|  | 9881967 |
|  | 9885919 |
|  | 9916716 |
|  | 9973388 |
|  | 10227975 |
|  | 10699178 |
|  | 10725748 |
|  | 10753117 |
|  | 11003391 |
|  | 11149986 |
|  | 11278629 |
|  | 11396683 |
|  | 11709756 |
|  | 11786536 |
|  | 11825773 |
|  | 11830476 |
|  | 11940068 |
|  | 12063528 |
|  | 12067309 |
|  | 12097410 |
|  | 12145428 |
|  | 12152987 |
|  | 12163193 |
|  | 12165514 |
|  | 12189139 |
|  | 12207328 |
|  | 12223527 |
|  | 12370393 |
|  | 12370400 |
|  | 12377947 |
|  | 12396617 |
|  | 12413765 |
|  | 12441140 |
|  | 12477932 |
|  | 12525574 |
|  | 12548511 |
|  | 12594065 |
|  | 12654630 |
|  | 12682264 |
|  | 12704784 |
|  | 12748293 |
|  | 12748907 |
|  | 12794003 |
|  | 12884288 |
|  | 12897746 |
|  | 12920029 |
|  | 12928861 |
|  | 12935900 |
|  | 1408833 |
|  | 14512310 |
|  | 14561191 |
|  | 14564352 |
|  | 14634100 |
|  | 14734771 |
|  | 14756548 |
|  | 14764428 |
|  | 14983029 |
|  | 14984938 |
|  | 15004182 |
|  | 15007352 |
|  | 15014952 |
|  | 15146247 |
|  | 15155567 |
|  | 15155645 |
|  | 15163701 |
|  | 15163899 |
|  | 15187156 |
|  | 15191916 |
|  | 15356556 |
|  | 15356573 |
|  | 15361241 |
|  | 15474066 |
|  | 15488706 |
|  | 15528258 |
|  | 15634905 |
|  | 15642470 |
|  | 15661077 |
|  | 15699166 |
|  | 15711639 |
|  | 15845862 |
|  | 15869882 |
|  | 15870068 |
|  | 15879126 |
|  | 15919378 |
|  | 15951665 |
|  | 16001979 |
|  | 16024972 |
|  | 16034134 |
|  | 16101942 |
|  | 16210662 |
|  | 16236836 |
|  | 16303333 |
|  | 16388492 |
|  | 16428771 |
|  | 16461126 |
|  | 16751012 |
|  | 16832637 |
|  | 16849505 |
|  | 16918506 |
|  | 16951338 |
|  | 16987060 |
|  | 17016624 |
|  | 17082655 |
|  | 17090525 |
|  | 17142763 |
|  | 17183946 |
|  | 7521694 |
|  | 7721105 |
|  | 8097324 |
|  | 8605348 |
|  | 9139718 |
|  | 9177784 |
|  | 9856949 |
|  | 9856950 |
|  | 10436181 |
|  | 10614495 |
|  | 10651852 |
|  | 11197307 |
|  | 11588017 |
|  | 16133987 |
|  | 17006604 |
|  | 17091279 |
|  | 17097743 |
|  | 17182591 |
|  | 17235327 |
|  | 17264150 |
|  | 17313976 |
|  | 17347445 |
|  | 17418380 |
|  | 17451413 |
|  | 17548690 |
|  | 17560640 |
|  | 17561245 |
|  | 17603012 |
|  | 17644317 |
|  | 7530359 |
|  | 10861042 |
|  | 12218103 |
|  | 12642601 |
|  | 16210602 |
|  | 16275384 |
|  | 16488622 |
|  | 16606668 |
|  | 17101709 |
|  | 17182558 |
|  | 17241396 |
|  | 17307869 |
|  | 17318894 |
|  | 17389581 |
|  | 17400600 |
|  | 17412309 |
|  | 17556359 |
|  | 17611223 |
|  | 17429054 |
|  | 17665443 |

---

|  |  |
| --- | --- |
| Organism | Homo sapiens {Organism urn:agi-taxid:9606} |
|  | Mus musculus {Organism urn:agi-taxid:10090} |
|  | Rattus norvegicus {Organism urn:agi-taxid:10116} |
|  | Mus musculus |
|  | Homo sapiens |
|  | Rattus norvegicus |
|  | Bos taurus |

---

|  |  |
| --- | --- |
| Mouse chromosome position | 11 31.98 cM |
|  | 11 29.0 cM |

---

|  |  |
| --- | --- |
| OMIM ID | 147683 |
|  | 607154 |
|  | 600807 |

---

|  |  |
| --- | --- |
| Rat chromosome position | 10q22 |

---

|  |  |
| --- | --- |
| Hugo ID | 5973 |
|  | HGNC:5973 |

---

|  |  |
| --- | --- |
| Human chromosome position | 5q31.1 |
|  | 5q31 |

---

|  |  |
| --- | --- |
| Swiss-Prot Accession | P35225 |
|  | P35225.2 |
|  | P20109 |
|  | P20109.1 |
|  | P42203 |
|  | P42203.1 |
|  | O43644 |
|  | Q4VB52 |
|  | Q9UDC7 |
|  | Q5SUZ9 |
|  | Q4VB50 |
|  | Q4VB51 |
|  | Q4VB53 |

---

|  |  |
| --- | --- |
| PIR ID | I38060 |
|  | E30552 |
|  | I52290 |

---

|  |  |
| --- | --- |
| GenBank ID | NC\_000005 |
|  | NM\_001354992 |
|  | NP\_001341921 |
|  | NM\_001354993 |
|  | NP\_001341922 |
|  | NM\_001354991 |
|  | NP\_001341920 |
|  | NM\_002188 |
|  | NP\_002179 |
|  | NG\_012090 |
|  | AC004039 |
|  | AC004041 |
|  | AF172149 |
|  | AF172150 |
|  | AF193838 |
|  | AF193839 |
|  | AF193840 |
|  | AF377331 |
|  | AAK53823 |
|  | AF416600 |
|  | AY008331 |
|  | AY008332 |
|  | CH471062 |
|  | EAW62324 |
|  | EAW62325 |
|  | EAW62326 |
|  | EAW62327 |
|  | HI573912 |
|  | CBX47445 |
|  | L13029 |
|  | L42079 |
|  | L42080 |
|  | U10307 |
|  | AAA83738 |
|  | U31120 |
|  | AAB01681 |
|  | AF043334 |
|  | AAC03535 |
|  | BC096138 |
|  | AAH96138 |
|  | BC096139 |
|  | AAH96139 |
|  | BC096140 |
|  | AAH96140 |
|  | BC096141 |
|  | AAH96141 |
|  | L06801 |
|  | AAA36107 |
|  | X69079 |
|  | CAA48823 |
|  | CAA48824 |
|  | P35225 |
|  | NC\_000077 |
|  | NM\_008355 |
|  | NP\_032381 |
|  | AC005742 |
|  | AL645741 |
|  | CH466575 |
|  | EDL33575 |
|  | L13028 |
|  | AA646049 |
|  | M23504 |
|  | AAA40149 |
|  | P20109 |
|  | NC\_005109 |
|  | NM\_053828 |
|  | NP\_446280 |
|  | AC\_000078 |
|  | AAHX01063901 |
|  | AC107611 |
|  | CH473948 |
|  | EDM04394 |
|  | L26913 |
|  | AAA16478 |
|  | P42203 |
|  | NC\_018916 |
|  | AMYH02012219 |
|  | AC\_000033 |
|  | AAHY01093363 |
|  | BC152854 |
|  | AAI52855 |
|  | AC\_000137 |
|  | CAI24689 |
|  | NT\_034772 |
|  | NW\_001838952 |
|  | NW\_922784 |
|  | AC\_000048 |
|  | Q4VB50 |
|  | Q4VB51 |
|  | Q4VB53 |
|  | NT\_096135 |
|  | NW\_001030461 |
|  | Q5SUZ9 |
|  | NW\_047334 |
|  | NW\_001084656 |
|  | Q4VB52 |
|  | NT\_039530 |
|  | NT\_086681 |
|  | 1170528 |
|  | 438876 |
|  | 129377 |
|  | 533247 |
|  | 56206964 |
|  | 81889541 |
|  | 1045452 |
|  | 1127548 |
|  | 116138167 |
|  | 121948508 |
|  | 14091716 |
|  | 186276 |
|  | 2905620 |
|  | 462408 |
|  | 580330 |
|  | 64653427 |
|  | 64654183 |
|  | 64654580 |
|  | 673420 |
|  | 74753700 |
|  | 74753701 |
|  | 74753702 |
|  | 4558814 |
|  | 5420145 |
|  | 7387804 |
|  | AAD22748 |
|  | AF072807 |
|  | AF440369 |
|  | AJ132441 |
|  | CAB46636 |
|  | NC\_007305 |
|  | NM\_174089 |
|  | NP\_776514 |
|  | NW\_001495335 |
|  | Q9XSV9 |
|  | 119582728 |
|  | 119582729 |
|  | 119582730 |
|  | 119582731 |
|  | 148701628 |
|  | 157170028 |
|  | 149052577 |
|  | ABBA01068803 |

---

|  |  |
| --- | --- |
| Swiss-Prot ID | IL13\_HUMAN |
|  | IL13\_MOUSE |
|  | IL13\_RAT |
|  | Q5SUZ9\_MOUSE |
|  | Q4VB52\_HUMAN |

---

|  |  |
| --- | --- |
| Cell Localization | Secreted |
|  | Extracellular |

---

|  |  |
| --- | --- |
| Ensembl ID | ENSG00000169194 |
|  | ENSP00000304915.3 |
|  | ENST00000304506.7 |
|  | ENSMUSG00000020383 |
|  | ENSMUSP00000020650.1 |
|  | ENSMUST00000020650.1 |
|  | ENSRNOG00000007652 |
|  | ENSRNOP00000010121.1 |
|  | ENSRNOT00000010121.2 |
|  | ENSP00000304915 |
|  | ENST00000304506 |
|  | ENSMUSP00000020650 |
|  | ENSMUST00000020650 |
|  | ENSRNOP00000010121 |
|  | ENSRNOT00000010121 |

---

|  |  |
| --- | --- |
| MGI ID | MGI:96541 |
|  | 96541 |

---

|  |  |
| --- | --- |
| RGD ID | 68949 |

---

|  |  |
| --- | --- |
| Unigene ID | Hs.845 |
|  | Mm.1284 |
|  | Rn.9921 |
|  | Bt.12928 |

---

|  |  |
| --- | --- |
| Homologene ID | 1649 |

---

|  |  |
| --- | --- |
| Shape | Rhomb |

---

|  |  |
| --- | --- |
| IPI ID | IPI00807607 |
|  | IPI00116193 |
|  | IPI00194737 |

---

|  |  |
| --- | --- |
| Microarray ID | 94168\_at |

---

|  |  |
| --- | --- |
| Homo sapiens Chromosome position | 5q31 |

---

|  |  |
| --- | --- |
| Ensembl Transcript ID | ENST00000304506 |

---

|  |  |
| --- | --- |
| FunctionalClass | signal transducer |
|  | cytokine |
|  | hematopoietin/interferon-class (D200-domain) cytokine receptor binding |
|  | interleukin-13 receptor binding |
|  | chemokine |

---

|  |  |
| --- | --- |
| Bos taurus Chromosome position | 7 |

---

|  |  |
| --- | --- |
| Mus musculus Chromosome position | 11 29.0 cM |

---

|  |  |
| --- | --- |
| KEGG pathway | Cytokine-cytokine receptor interaction |
|  | Jak-STAT signaling pathway |
|  | Fc epsilon RI signaling pathway |

---

|  |  |
| --- | --- |
| Rattus norvegicus Chromosome position | 10q22 |

---

|  |  |
| --- | --- |
| Ensembl Gene ID | ENSG00000169194 |

---
